# Divergent Immunomodulatory Roles of Fungal DNA in Shaping Treg and Inflammatory Responses

**DOI:** 10.3390/jof11110760

**Published:** 2025-10-22

**Authors:** Dongmei Li, Idalia Cruz, Yahui Feng, Maha Moussa, Jie Cheng, Digvijay Patil, Alexander Kroemer, Joseph A. Bellanti

**Affiliations:** 1 Department of Microbiology & Immunology, Georgetown University Medical Center, Washington, DC 20057, USA; 2 Department of Oncology, Animal Models, Shared Resources, Georgetown University Medical Center, Washington, DC 20057, USA; 3 Laboratory of Medical Mycology, Jining No. 1 People’s Hospital, Jining 272067, China; 4 MedStar Georgetown Transplant Institute, MedStar Georgetown University Hospital and the Center for Translational Transplant Medicine, Georgetown University Medical Center, Washington, DC 20057, USA; 5 Department of Pediatrics, Georgetown University Medical Center, Washington, DC 20057, USA

**Keywords:** fungal DNA, DNA sensing, Treg, IL-10, TLR-9, cGAS-STING pathway

## Abstract

Fungal communities in the gut influence host immunity, yet most studies have focused on cell wall components rather than genetic materials. Here, we explore how fungal genomic DNA (gDNA) from *Candida albicans*, *Saccharomyces cerevisiae*, and *Cryptococcus neoformans* modulate immune responses in human CD4^+^ T cells, murine splenocytes, and THP-1-derived macrophages. We find that *C. albicans* gDNA promotes the development of regulatory T cells and increases IL-10, fostering immune tolerance and preserving CD4^+^ T cell viability in an inflammatory setting. *S. cerevisiae* gDNA induces moderate Treg responses with restrained effector T cell expansion and higher checkpoint gene expression, entirely consistent with its commensal nature. In contrast, *C. neoformans* gDNA elicits a strongly inflammatory profile, promoting Th1/Th17 cells and driving high cytokine production. Mechanistically, *C. albicans* and *S. cerevisiae* gDNA dampen DNA-sensing pathways and enhance immune checkpoint molecules that act as brakes against overactivation, while *C. neoformans* gDNA robustly activates innate sensing pathways with limited checkpoint induction. These species-specific signaling profiles reveal that fungal gDNA itself can influence whether the immune system adopts a tolerant or inflammatory response toward fungi. This discovery highlights fungal genomic DNA as a previously underappreciated regulator of host–fungus interactions, offering new insight into commensal persistence, pathogenic invasion, and the potential for DNA-based antifungal interventions.

## 1. Introduction

Fungal DNA is usually considered to be quite similar to that of other eukaryotic organisms, in that it is normally confined to the nucleus and mitochondria. However, during host–fungus interactions, whether in a commensal or invasive state, fungal nucleic acids can be released into the surrounding environment. First, nucleic acids may be passively liberated through immune system activity, such as through neutrophil extracellular traps (NETs) [1,2], by means of macrophage-mediated killing, or through fungal cell lysis triggered by antifungal exposure or reactive oxygen species (ROS) in hostile environments [3]. Second, fungi can actively secrete DNA via extracellular vesicles [4] or incorporate it into biofilm structures [5]. While mammalian and bacterial nucleic acids are increasingly recognized as potent immunostimulatory signals [6,7,8], the immunological role of fungal-derived DNA remains largely unexplored.

Research on fungal immune activation has predominantly emphasized cell wall components, including mannoproteins and glucans from *Candida albicans* [9,10], which function as pathogen-associated molecular patterns (PAMPs) or damage-associated molecular patterns (DAMPs). These ligands are recognized by pattern recognition receptors (PRRs) expressed on the surface of host immune cells, thereby initiating a critical first line of defense against fungal invasion. However, while effective antifungal immune responses shape the progression and severity of fungal infections [11,12,13], if these immune responses are not themselves properly regulated, they can also contribute to the exacerbation of chronic inflammation–related diseases such as psoriasis [14].

To suppress excessive inflammation and maintain a balanced immune environment, the abundance of regulatory T (Treg) cells is now [15,16,17] recognized as a key determinant in various inflammatory conditions, including allergic disorders, chronic inflammation, and cancer, where it directly influences disease development and progression. Beyond the well-established roles of classical PAMPs, accumulating evidence indicates that microbial DNA also functions as a potent danger signal capable of activating innate immune pathways [7,8,18,19] and promoting Treg expansion [20]. In our recent study [21], we demonstrated that probiotic bacterial DNA helps to maintain immune homeostasis by promoting Treg expansion and enhancing secretion of the anti-inflammatory cytokine interleukin-10 (IL-10). These findings prompt us to hypothesize that fungal genomic DNA (gDNA) may similarly act as an immunomodulatory signal that influences the balance between immune activation and tolerance.

Regulatory T cells (Tregs) are critical for sustaining immune tolerance and limiting excessive inflammation. They mediate suppression through cell-contact mechanisms and the secretion of anti-inflammatory cytokines, including IL-10 and TGF-β [22,23]. Defined by CD4 and CD25 co-expression and high Foxp3 levels [24], Tregs restrain CREB/ATF- and NF-κB–driven inflammatory pathways [25]. Their development and stability depend on complex transcriptional and epigenetic regulation [26,27,28]. Given their central role in immune regulation, investigating how different fungal DNAs (commensal versus pathogen) influence Treg-mediated immune balance represents an important step toward understanding host–fungus interactions.

*C. albicans* is a natural component of the human gastrointestinal microbiota [29,30]. However, under certain conditions, it can turn from a commensal organism into an invasive pathogen in susceptible individuals [31,32]. Host defense against fungal pathogens relies heavily on CD4^+^ T helper 1 (Th1) and Th17 cell subsets, which promote fungal clearance through the production of proinflammatory cytokines that enhance phagocytic activity [33]. While such responses are essential for pathogen elimination, they carry the risk of excessive inflammation and collateral tissue damage if not properly regulated [34,35,36,37]. This delicate balance between antifungal activation and immune tolerance suggests that *C. albicans*, as an opportunistic organism, may elicit dual immune traits—promoting sufficient immune activation to control fungal growth while simultaneously preserving tissue homeostasis. Given our previous findings that probiotic bacterial DNA promotes Treg expansion and anti-inflammatory cytokine production [21], we hypothesize that *C. albicans* genomic DNA (gDNA) might similarly contribute to maintaining immune balance.

To test this hypothesis, we investigate the immunomodulatory potential of *C. albicans* gDNA on human CD4^+^ T cells and murine splenocytes, focusing on Treg induction and cytokine secretion profiles. To determine whether these effects are species-specific or conserved across fungi with different lifestyles, we compare *C. albicans* gDNA with that of *Saccharomyces cerevisiae* (a non-pathogenic yeast) and *Cryptococcus neoformans* (a pathogenic fungus). In parallel we examine innate immune signaling using THP-1–derived macrophages. Together, these experiments reveal that fungal genomic DNA functions not merely as a structural component (e.g., in biofilm formation) but as a potential immunoregulatory molecule shaping host immune responses.

## 2. Materials and Methods

### 2.1. Fungal Strain and DNA Extraction

The extraction of genomic DNA was performed following established protocols from pure fungal cultures [38]. Three fungal species (all wild type)—*Candida albicans* SC5314, *Cryptococcus neoformans* var. *neoformans* JEC21, and *Saccharomyces cerevisiae* BY4742 (derived from wild-type S288C with lysine auxotrophic)—were included in this experiment. These fungal isolates were maintained at −80 °C until use. Prior to DNA extraction, each isolate was grown in YPD broth medium (1% yeast extract, 2% peptone and 2% dextrose) overnight at 30 °C, with 200 rpm shaking. Then, an RNAase treatment at 37 °C for 2 h was applied to each DNA sample to eliminate RNA interference in subsequent experiments. DNA concentration and purity were assessed using a nanodrop 2000C spectrophotometer (Thermo Scientific, Waltham, MA, USA), with a target 260/280 ratio ranging from 1.8 and 2.0.

### 2.2. Reagents Used in Human PBMC-iTreg Experiment

The following reagents were used in the human PBMC-iTreg portion of the experiment. We used Ficoll-Paque™ Plus (Amersham Pharmacia Biotech AB, Uppsala, Sweden) for density gradient separation and CellometerViaStain™ AOPI staining solution for cell viability assessment. CD4^+^ T cells were isolated using the Human CD4^+^ T Cell Isolation Kit and MiniMACS separation columns (MiltenyiBiotec, Cologne, Germany). Purified human immunoglobulin IgG (Sigma, St. Louis, MO, USA) was used during cell preparation, and X-VIVO™ 15 serum-free hematopoietic cell medium (Lonza, Basel, Switzerland) was used for culture. Recombinant human interleukin-2 (IL-2) and transforming growth factor-beta (TGF-β) were obtained from R&D Systems (Minneapolis, MN, USA) to support Treg induction. For flow cytometry, Live/Dead staining reagents were purchased from Invitrogen (Carlsbad, CA, USA) and FACS buffer was prepared with PBS containing either 0.5–1% BSA or 5–10% FBS, and 0.1% sodium azide (eBioscience/Invitrogen, Carlsbad, CA, USA). T cell stimulation was achieved using purified NA/LE mouse anti-human CD3 (Clone: UCHT1) and anti-human CD28 (Clone: CD28.2) antibodies (BD Biosciences, Franklin Lakes, NJ, USA). For surface and intracellular staining, we used a FOXP3 fixation/permeabilization buffer set (BioLegend, San Diego, CA, USA), along with the following fluorochrome-conjugated monoclonal antibodies: Brilliant Violet 421 anti-human CD4 (Clone: A161A1), APC anti-human CD25 (Clone: BC96), and PE anti-human Foxp3 (Clone: 206D), all from BioLegend.

### 2.3. Human PBMC CD4^+^ Isolation

Human PBMCs were obtained from healthy donors (n = 3) under Institutional Review Board–approved protocol # STUDY00001964 (Georgetown University). Written informed consent was obtained from all donors in the experiments described previously [20], where we included assays using *Bifidobacterium* DNA, *E. coli* DNA, and other microbial DNA stimuli [27]. In brief, 20 mL of EDTA-treated blood was used to isolate PBMC using Ficoll-Paque™ Plus (Amersham Pharmacia Biotech AB). After washing with 0.15 M saline, CD4^+^ T cells were isolated using the Human CD4^+^ T Cell Isolation Kit and MiniMACS separation columns (MiltenyiBiotec), with the separation process carried out on the AutoMACS magnetic separation system. Cell viability and concentration were stained using ViaStain™ AO/PI staining and analyzed with the Cellometer Auto 2000 (Nexcelom Bioscience LLC, Lawrence, MA, USA). The purified CD4^+^ T cells were then re-suspended in X-VIVO 15 serum-free hematopoietic medium (Lonza) at a concentration of 2 × 10^6^ cells/mL.

### 2.4. Induction of iTreg (Induced Tregs) In Vitro by C. albicans gDNA

**Figure 1:**
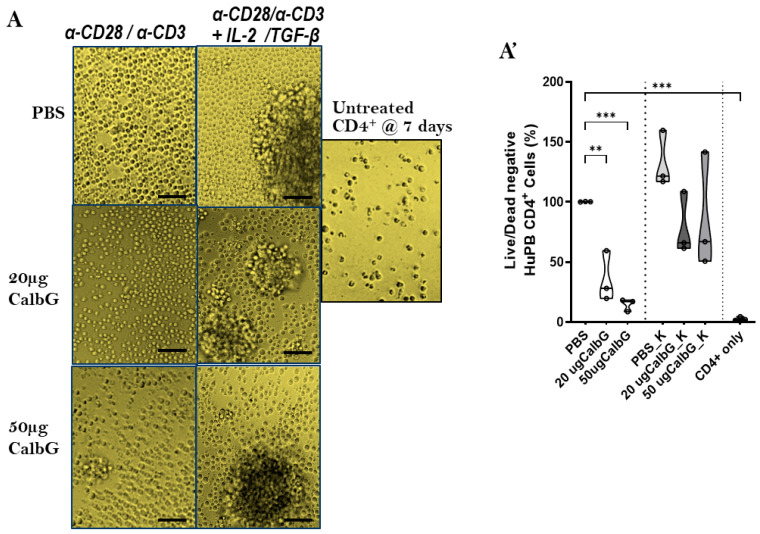
*Candida albicans* gDNA impairs survival of activated CD4^+^ T cells rescued by IL-2/TGF-β. (**A**) Representative phase-contrast micrographs of human PBMC-derived CD4^+^ T cells cultured for 7 days under the indicated conditions. Cells were activated with anti-CD3/CD28 in PBS (control), 20 µg or 50 µg *C. albicans* gDNA (CalbG), or co-treated with IL-2/TGF-β (_K conditions). Un-stimulated CD4^+^ T cells (“CD4^+^ only”) were maintained for the same 7 days (@_)_ duration. Scale bars: 100 µm. (**A′**) Quantification of viable CD4^+^ T cells (Live/Dead negative) expressed as a percentage relative to PBS-treated controls. Open circles indicate mean values of duplicate measurements per donor. HuPB: Human peripheral blood. Data from three healthy donors are shown as mean ± SD; *p* < 0.01 (**), and *p* < 0.001 (***) using unpaired Student’s t-test and one-way ANOVA.

To induce Treg cells in vitro, a 96-well flat-bottom plate was prepared by pre-coating each well with 50 µL PBS containing 10 µg/mL anti-human CD3 (α-CD3) and anti-human 5 µg/mL anti-human CD28 (α-CD28) antibodies, and incubated overnight at 4 °C. As a methodological control, some wells were coated with PBS alone (untreated CD4^+^ in Figure 1A). After removing the coating solution and washing the wells twice with PBS, 150 µL of purified CD4^+^ T cells (3 × 10^5^ cells per well) were added. *C. albicans* genomic DNA (CalbG in Figure 1A) was then added either alone or in combination with Treg-inducing cytokines—recombinant human IL-2 (60 IU) and TGF-β (50 IU)—in 50 µL of X-VIVO 15 serum-free medium. Cells were cultured at 37 °C in a 5% CO_2_ incubator.

Based on preliminary PBMC experiments testing 5–100 µg of DNA, 25 µg was selected for subsequent experiments, as lower doses (<20 µg) had no effect and higher doses (>50 µg) caused T cell loss likely due to cytotoxicity. On day 4, half of the culture in each stimulated well was transferred into a new 96-well plate pre-coated with the same α-CD3 and α-CD28. The wells in both plates were then topped up to a final volume of 200 µL with fresh X-VIVO 15 medium and incubated under the same conditions for an additional 3 days.

### 2.5. Human CD4^+^ T Cell Staining and FACS Analysis

On day 7 of culture, stimulated CD4^+^ T cells were harvested and stained in polystyrene round-bottom 12 × 75 mm BD Falcon tubes, protected from light, at 4 °C for 30 min. After two washes with cold PBS by centrifuging at 1500 rpm for 5 min at 4 °C, cells were first stained with a Live/Dead viability dye, followed by additional washes with PBS and FACS buffer. To block non-specific Fc receptor binding, cells were incubated with 10 µL of human IgG (1 mg/mL) at 4 °C for 10 min. Surface staining was then performed using a 100 µL cocktail of FACS buffer containing Brilliant Violet 421 anti-human CD4 and APC anti-human CD25 antibodies. After two washes with FACS buffer, cells were fixed and permeabilized using the Foxp3 Fixation Buffer at 4 °C for 45 min.

Intracellular staining was performed by incubating cells in 100 µL of Perm/Wash buffer containing PE-conjugated anti-human FoxP3 antibody. Following an additional wash with Perm/Wash buffer, stained cells were re-suspended in 200 µL of FACS buffer for flow cytometric analysis on a BD FACS Symphony instrument. Data were analyzed using FlowJo software version 10 (Ashland, OR, USA). A full minus-one (FMO) control was included in parallel to aid in the gating of CD25^+^FoxP3^+^ Treg cell populations (Supplemental Figure S1).

### 2.6. Reagents Used in Mouse Splenocytes-Treg Experiment

To characterize CD4^+^ T cell subsets (including Treg, Th1, Th2, and Th17 populations), B cells, and overall cell viability in mouse splenocyte cultures, we performed multicolor flow cytometric analysis using a panel of fluorescently conjugated anti-mouse antibodies and viability dyes [21]. All reagents were used according to the manufacturers’ protocols and recommended concentrations. The following antibodies were purchased from BioLegend: BV605 anti-CD3 (clone 17A2, cat# 100237), BV785 anti-CD4 (clone GK1.5, cat# 100453), APC/Cyanine7 anti-CD25 (clone PC61.5, cat# 102025), BV421 anti-T-bet (clone 4B10, cat# 644832), PerCP/Cy5.5 anti-GATA3 (clone TWAJ, cat# 653811), Alexa Fluor^®^ 488 anti-Foxp3 (clone MF-14, cat# 126405), PE/Dazzle™ 594 anti-IFN-γ (clone XMG1.2, cat# 505845), BV711 anti-IL-4 (clone 11B11/BVD6-24G2, cat# 504133), PE anti-IL-17A (clone TC11-18H10.1, cat# 506903), APC anti-IL-10 (clone JES5-16E3, cat# 505009), and BV510 anti-B220/CD45R (clone RA3-6B2, cat# 103248). In addition, PE-Cyanine7 anti-RORγt (clone B2D, cat# 25-6981-80) was obtained from eBioscience. Zombie Violet viability dye (BioLegend) was used to exclude non-viable cells.

### 2.7. Mouse Splenocyte Preparation

Mouse splenocytes were obtained from control animals from our previous study conducted under protocol #2022-0021, approved by the Institutional Animal Care and Use Committee (IACUC) of Georgetown University [39]. These mice received two intraperitoneal injections of PBS over a 17-day period. Spleens were mechanically dissociated by gently pressing through a 70 µm cell strainer. Splenocytes pooled from three mice were washed twice with RPMI-1640 medium and re-suspended in 10 mL of RPMI-1640 supplemented with 10% fetal bovine serum (FBS).

Flat-bottom 96-well plates were pre-coated overnight at 4 °C with 50 µL PBS containing 10 µg/mL anti-CD3 antibody (Purified NA/LE Hamster Anti-Mouse CD3ε, BD Pharmingen™). Splenocytes were seeded at 2 × 10^6^ cells per well in RPMI-1640 medium with 10% FBS. Genomic DNA (25 µg) from each yeast species was added to the appropriate wells, along with 50 IU each of recombinant IL-2 and TGF-β to support T cell activation and Treg induction. Plates were incubated at 37 °C in a 5% CO_2_ atmosphere, and T cell responses were evaluated by flow cytometry after 24 h of culture.

### 2.8. Fluorescent Staining of Splenocytes and Flow Cytometric Analysis

Fluorescent staining and flow cytometry were performed following our protocols [40]. Treated splenocytes were pelleted by centrifugation at 400~600× *g* for 5 min at 4 °C, washed with Dulbecco’s phosphate-buffered saline (DPBS, without calcium and magnesium), and stained with 100 µL Zombie Violet viability dye (1:500 in DPBS) for 15 min at room temperature. Parallel Zombie-only and no-Zombie controls were included to facilitate viable cell gating (Supplemental Figure S2).

After washing with DPBS, cells were stained with a surface antibody cocktail containing fluorescently labeled anti-CD3, anti-CD4, anti-CD25, and anti-CD220 in staining buffer for 20 min at room temperature in the dark. Fluorescence minus one (FMO) controls were included for each marker. Cells were then washed twice with staining buffer, fixed with 1× Fixation Buffer (True-Nuclear™ Fix Diluent, BioLegend) for 45 min, and permeabilized with Perm Buffer for 20 min. Intracellular staining was performed by incubating cells with a cocktail of antibodies against T-bet, GATA3, RORγt, FOXP3, IL-4, IL-17A, and IFN-γ in Perm Buffer for 15 min at room temperature in the dark. Corresponding FMOs were prepared for each intracellular marker. Following a final wash with Perm Buffer, cells were re-suspended in FACS staining buffer for acquisition.

Data were collected on a BD Fortessa SORP flow cytometer (BD Biosciences) and analyzed using FCS Express 7.24.0030 (DeNovo Software, Pasadena, CA, USA). Compensation was performed using antibody capture beads (BioLegend). T cell subsets were analyzed from gated viable CD3^+^CD4^+^ cells, including Tregs (CD25^+^FOXP3^+^), Th1 (IFN-γ^+^T-bet^+^), Th2 (IL-4^+^GATA3^+^), and Th17 (IL-17A^+^RORγt^+^). IL-10^+^ B cells (regulatory B cells, Bregs) were defined as CD220/CD45R^+^IL-10^+^ gated within CD220/CD45R^+^CD3^−^ cells (Supplemental Figure S2).

### 2.9. RNA Isolation and Real-Time PCR Analysis

Total RNA was extracted from THP-1–derived macrophages 3 h after fungal DNA stimulation using TRIzol (Invitrogen). cDNA was synthesized with a reverse transcription kit (Thermo Fisher Scientific) and subjected to RT-qPCR using SYBR Green Master Mix on a Bio-Rad CFX96 system. Expression of *TLR-2*, *TLR-4*, *TLR-7*, *TLR-8*, *TLR-9*, *CTLA-4*, *LAG3*, *PD-1*, and *IDO-1* was quantified with primers (Table S1) and normalized to GAPDH using the 2^−ΔΔCt^ method.

### 2.10. Western Blot Analysis of THP-1-Derived Macrophages Exposed to Different Fungal DNA

THP-1 monocytes (ATCC) were cultured in RPMI-1640 supplemented with 10% fetal bovine serum and 1% penicillin–streptomycin at 37 °C, 5% CO_2_. Cells were differentiated into macrophage-like cells by seeding at 1 × 10^6^ cells/mL and treating with 100 pg/mL PMA for 48 h, followed by a 24 h rest in PMA-free medium. Differentiated macrophages were stimulated with medium alone (negative control), LPS (0.1 ng/mL; Sigma), 25 μg genomic DNA (gDNA) from *Candida albicans* (CalbG), *Saccharomyces cerevisiae* (ScerG), or *Cryptococcus neoformans* (CrypG).

Protein lysates were collected 6 h post-treatment using pre-cooled RIPA buffer with protease and phosphatase inhibitors [41]. Equal protein amounts were resolved by SDS-PAGE, transferred to PVDF membranes, and probed with primary antibodies (Cell Signaling Technology, Danvers, MA, USA) against cGAS, phospho-STING (p-STING), phospho-TBK1 (p-TBK1), phospho-IRF3 (p-IRF3), TLR-9, TRAF-6, and GAPDH (Cat. no. 5174), followed by HRP-conjugated secondary antibodies. Bands were visualized using ECL and quantified with ImageJ v6.0.

### 2.11. Statistical Analysis

Unpaired Student’s t-tests were used to assess statistical significance for iTreg (CD25^+^FoxP3^+^CD4^+^) frequencies and IL-10 levels when comparing two groups. For experiments involving multiple groups, statistical significance was assessed using either one-way ANOVA with Benjamini–Hochberg false discovery rate (FDR) correction or two-way ANOVA with Dunnett’s post hoc test, depending on the experimental design. Data obtained from biological triplicates are expressed as the mean ± standard deviation (SD). Statistical significance was defined as *p* < 0.05. All analyses were performed using GraphPad Prism v4 (GraphPad Software, San Diego, CA, USA).

## 3. Results

### 3.1. Candida albicans gDNA Impairs Survival of Activated CD4^+^ T Cells Rescued by IL-2/TGF-β

To determine whether fungal DNA directly affects CD4^+^ T cell fitness, we examined the impact of *C. albicans* gDNA (CalbG) on human peripheral blood CD4^+^ T cells activated with anti-CD3/CD28. By day 7, CD4^+^ T cells stimulated with anti-CD3/CD28 (PBS condition) showed a modest increase compared to un-stimulated controls (CD4^+^ only, *p* < 0.001; Figure 1A,A’). In contrast, CalbG showed a markedly reduced CD4^+^ T cell increase in a dose-dependent manner, with a greater impairment at 50 µg than at 20 µg. CalbG-treated CD4^+^ T cells also exhibited pronounced morphological deterioration (Figure 1A).

Quantification of viable CD4^+^ T cells at day 7 is shown in Figure 1A’. At 50 µg CalbG, viability fell to <2%, comparable to un-stimulated CD4^+^ T cells. It was notable that co-treatment with IL-2 and TGF-β (K condition) preserved cell morphology and maintained viability at ~60–70% despite CalbG exposure. Although viability in CalbG_K remained lower than in PBS_K, it was not significantly different from the PBS condition without CalbG.

Together, these results demonstrate that CalbG selectively compromises activated CD4^+^ T cell expansion and viability, and that a regulatory cytokine milieu (IL-2/TGF-β) can counteract this effect. Given the critical role of CD4^+^ T cells in antifungal immunity, these findings prompted us to investigate whether the IL-2/TGF-β–induced regulatory T cell axis contributes to the protection of CD4^+^ T cell expansion and viability observed here.

### 3.2. IL-2/TGF-β Enables Candida gDNA-Driven iTreg Induction and IL-10 Production

**Figure 2:**
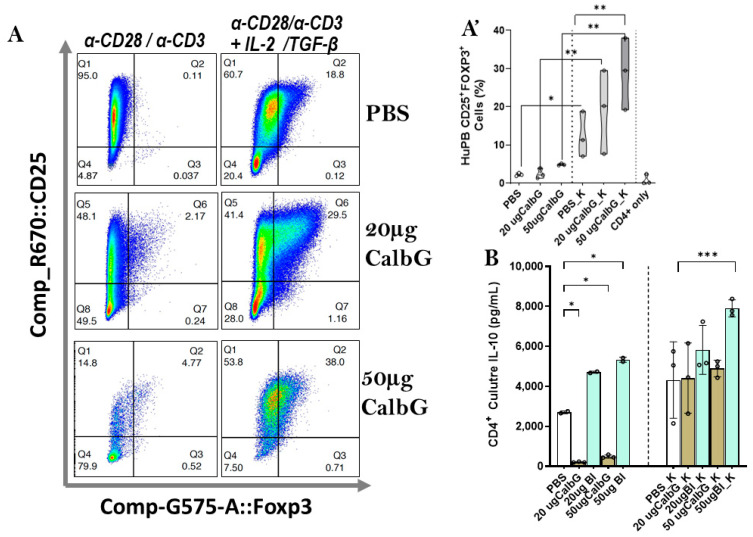
IL-2/TGF-β enables fungal DNA–driven iTreg induction and IL-10 production. (**A**) Representative flow cytometry plots showing CD25 and FoxP3 expression in PBMC-derived CD4^+^ T cells cultured for 7 days under the indicated conditions: PBS (control), *C. albicans* gDNA (CalbG; 20 or 50 µg), or *Bifidobacterium longum* DNA (BI; 20 or 50 µg), with or without IL-2/TGF-β (K conditions). (**A’**) Quantification of CD25^+^FoxP3^+^CD4^+^ T cells (%) from three independent donors. (**B**) IL-10 concentrations in culture supernatants measured by ELISA. Data are shown as mean ± SD; Open circles indicate the mean of duplicate measurements per experiment. Green and brown bars represent data from cultures treated with BI DNA and *C. albicans* gDNA, respectively. *p* < 0.05 (*), *p* < 0.01 (**), and *p* < 0.001 (***), using unpaired Student’s t-test or one-way ANOVA as appropriate.

Having established that *C. albicans* genomic DNA compromises the survival of activated human CD4^+^ T cells, we next investigated whether this effect involves induced regulatory T cells (iTregs; CD25^+^FoxP3^+^ CD4^+^ T cells) and their signature cytokine IL-10 under the same conditions described in Figure 1. Flow cytometry revealed that *C. albicans* genomic DNA (CalbG) alone slightly increased iTreg percentages, but the increase was not significant compared with PBS controls (Figure 2A,A’). However, when co-cultured with IL-2/TGF-β, both CalbG-treated groups showed significantly higher iTreg percentages than their counterparts without IL-2/TGF-β (*p* < 0.01). Notably, CalbG_K was also significantly higher than PBS_K (*p* < 0.05), indicating that fungal DNA possesses iTreg-inducing potential in a supportive cytokine milieu.

Consistent with the Treg response, IL-10 secretion was significantly increased in CalbG_K cultures (Figure 2B), reaching levels comparable to those induced by *Bifidobacterium longum* DNA (BI), a well-established IL-10 inducer from our earlier study [20]. In contrast, CalbG alone induced less IL-10 than the PBS control (*p* < 0.05), despite generating a small iTreg population. This finding indicates that fungal DNA by itself is insufficient to drive a robust regulatory phenotype. Instead, the presence of IL-2/TGF-β appears essential, converting fungal DNA stimulation from a detrimental signal into a protective one for CD4^+^ T cells by promoting iTreg differentiation and IL-10 production.

### 3.3. C. albicans gDNA Reduces Splenocyte Viability

To validate the iTreg induction observed with *C. albicans* DNA, we used an ex vivo murine splenocyte culture system to further examine Treg and effector T cell responses. Splenocytes, which contain diverse immune cell populations, provide an advantage over purified CD4^+^ T cell cultures by being able to capture interactions between antigen-presenting cells, T cells, and cytokine milieus that shape CD4^+^ activation and polarization. To determine whether the Treg effects of *C. albicans* genomic DNA (CalbG) are species-specific, we also included genomic DNAs isolated from non-pathogenic *Saccharomyces cerevisiae* and pathogenic *Cryptococcus neoformans* for comparison.

**Figure 3:**
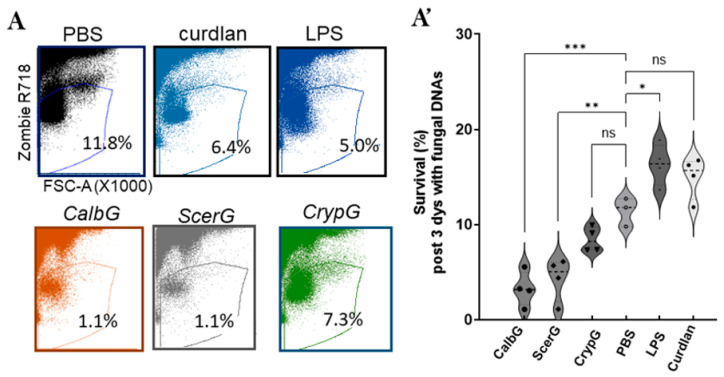
Effect of different fungal DNAs on murine splenocyte viability after 3 days of treatment. (**A**) Representative flow cytometry plots show forward scatter (FSC-A) gating of viable cells from splenocyte cultures treated with PBS, LPS, curdlan, and fungal DNA samples. Percentages indicate viable cell populations within each condition. (**A’**) Quantification of survival percentages post 3-day culture with gDNA from *C. albicans* (CalbG), *S. cerevisiae* (ScerG), and *C. neoformans* (CrypG) are presented. Open and closed symbols indicate the mean of duplicate measurements per experiment. Data represented as mean ± SD from four independent experiments. Statistical analysis from four independent experiments shows significant reductions in viability with each treatment compared to controls (* *p* < 0.05, ** *p* < 0.01, *** *p* < 0.001).

Splenocytes were isolated from wild-type mice and pretreated with DNase I to remove contaminating free DNA before stimulation with genomic DNA derived from *C. albicans*, *S. cerevisiae*, or *C. neoformans*. After three days of culture, cell viability was assessed by flow cytometry (Figure 3A,A’). Compared with the enhanced splenocyte viability observed in lipopolysaccharide (LPS)-stimulated cultures, fungal DNA treatment either reduced or maintained the proportion of viable cells relative to non-stimulated (PBS) controls. Consistent with the cytotoxic effects previously observed on CD4^+^ T cells (Figure 1), *C. albicans* gDNA, like *S. cerevisiae* gDNA, markedly decreased cell survival (~1.1% vs. 11.8% in PBS controls, *p* < 0.01). In contrast, *C. neoformans* genomic DNA supported modest survival (7.3%), which was not significantly different from PBS-treated splenocytes and resembled the pattern induced by curdlan, a fungal cell wall β-1,3-glucan mimic. These findings suggest that overall splenocyte viability reflects the balance between cell expansion, as observed under LPS stimulation, and the cytotoxic effects associated with fungal DNA. The relatively “lower toxicity” of *C. neoformans* DNA may reflect an immune cell expansion response, analogous to that triggered by microbial pattern recognition ligands such as LPS and curdlan [42,43,44].

### 3.4. CD4^+^ T Cell Subset Responses Reveal Divergent Effects of Fungal gDNA

As shown above, overall splenocyte viability reflected the interplay between immune cell expansion, as seen under LPS stimulation, and the cytotoxic effects associated with fungal DNA. To further dissect these outcomes, we next investigated whether this balance differentially affected CD4^+^ T cell subsets. Specifically, we examined whether the reduced viability observed under *C. albicans* genomic DNA (CalbG) was linked to impaired Treg responses, and whether the relatively higher survival under *C. neoformans* genomic DNA (CrypG) reflected selective expansion of effector populations. CD4^+^ T cell subsets were therefore compared across each fungal DNA treatment, with curdlan and LPS included as controls.

**Figure 4:**
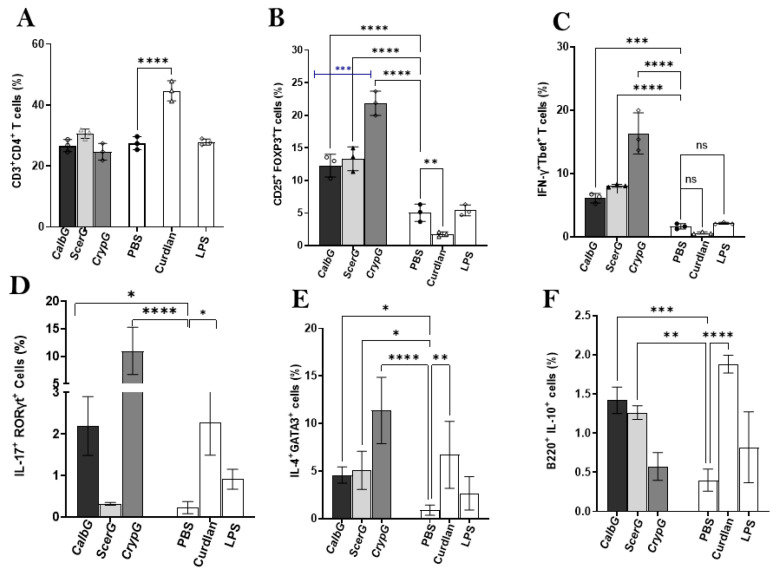
Differential immune cell subset responses to fungal DNA and curdlan in murine splenocytes. Splenocytes from wild-type mice were treated with PBS, curdlan, or fungal genomic DNAs from *C. albicans* (Calb), *S. cerevisiae* (Scer), or *C. neoformans* (Cryp) for 3 days, followed by flow cytometric analysis of immune cell subsets. (**A**) CD4^+^ T cell percentages were reduced by all DNA treatments compared with PBS, except for curdlan, which markedly increased CD4^+^ frequencies. (**B**) All fungal DNA preparations induced substantial Treg increases, most prominently with Cryp gDNA. (**C**) Th1 induction pattern was similar to Treg frequencies across all samples. (**D**) CalbG and CrypG showed elevated Th17 frequencies compared with PBS, whereas ScerG did not. (**E**) All three yeast gDNAs, particularly CrypG, significantly increased Th2 responses. (**F**) IL-10^+^ B cell frequencies were significantly increased by CalbG and ScerG, but not by CrypG. Data are shown as mean ± SD from three independent experiments. (* *p* < 0.05, ** *p* < 0.01, *** *p* < 0.001, and **** *p* < 0.0001).

All fungal gDNA preparations elicited moderate Treg increases, in contrast to the modest induction observed with curdlan (Figure 4B), despite its higher overall CD4^+^ proportions (Figure 4A). Notably, CrypG induced significantly higher Treg frequencies than CalbG (*p* < 0.001), consistent with its higher splenocyte viability (Figure 3A). For effector subsets, Th1 (IFN-γ^+^Tbet^+^) responses followed a similar pattern to Treg, with CrypG driving the strongest expansion (Figure 4C). Th17 (IL-17^+^RORγt^+^) frequencies were moderately elevated by CalbG, comparable to curdlan, but markedly increased by CrypG (Figure 4D). Th2 (IL-4^+^GATA3^+^) responses largely mirrored the Th17 pattern across all conditions, with the exception of ScerG, which induced significantly higher Th2 frequencies (Figure 4E). In contrast, IL-10^+^ B cells (Bregs; CD220^+^/CD45R^+^CD3^−^IL-10^+^) were more strongly induced by CalbG and ScerG, reaching levels comparable to curdlan (Figure 4F).

Together, these results reveal species-specific Treg and Teff subset responses across the three yeast DNAs. The Treg response appeared to be more closely associated with genomic DNA (gDNA) than with curdlan stimulation and showed a stronger alignment with Th1 responses. Elevated Treg responses to pathogenic *Cryptococcus* gDNA likely reflected the overall immunogenicity of its DNA, as demonstrated by the concurrent robust expansion of Th1, Th17, and Th2 subsets (Figure 4C–E), which may explain the relatively robust cell viability observed (Figure 3A). In contrast, CalbG and ScerG induced stronger IL-10^+^ B cell (Breg) responses alongside their modest increases in Treg populations, which corresponded to modestly elevated Th1 and Th2 subsets. It is also of some interest to note that Th2 frequencies were elevated across all tested fungal DNAs and curdlan, including the ScerG, whereas Th17 expansion was predominantly observed in the CalbG and CrypG groups, but not in ScerG. This generalized Th2 bias is consistent with prior observations that both commensal and pathogenic fungi tend to skew host immunity toward type 2 responses [45], a phenomenon thought to mirror antiparasitic defense, though only a subset of fungi ultimately manifest as clinical allergies.

### 3.5. THP-1 Macrophage mRNA Reveals Species-Specific TLR Activation and Checkpoint Gene Induction

**Figure 5:**
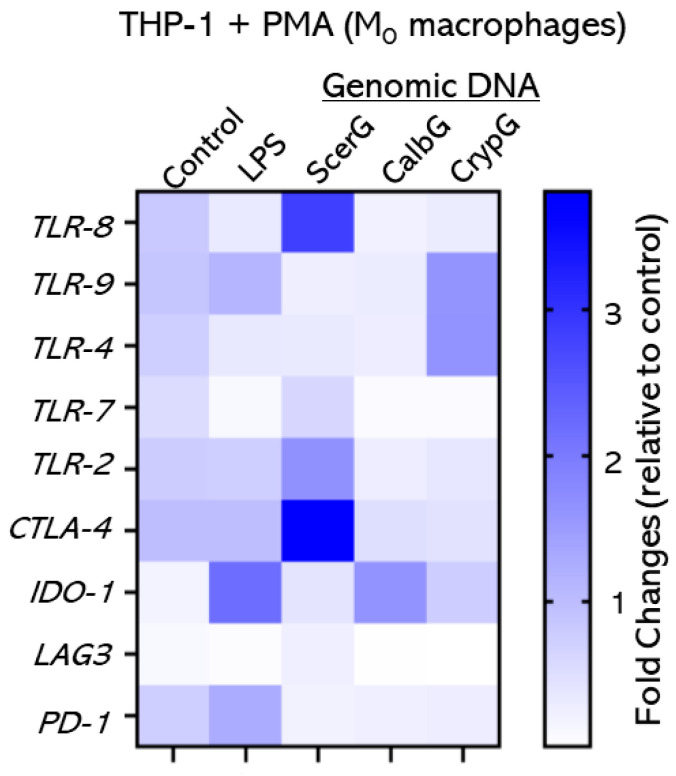
Distinct TLR and checkpoint molecule expression profiles in macrophages exposed to fungal DNAs. Macrophages were treated for 3 h with PBS (Control), LPS, or 25 µg of genomic DNA from *C. albicans* (CalbG), *S. cerevisiae* (ScerG), or *C. neoformans* (CrypG). mRNA levels of the indicated genes were measured by RT-qPCR and normalized to GAPDH in un-stimulated control macrophages. Data from three independent experiments are presented as a heatmap to show -fold changes relative to control. CalbG and ScerG downregulated or maintained most *TLR*s and uniquely increased *IDO-1* and *CTLA-4* expression, respectively. CrypG strongly upregulated *TLR-4* and, in particular, *TLR-9*, exceeding the levels induced by LPS.

To explore the molecular mechanisms by which fungal DNA modulates antigen-presenting cells (APCs) to influence Treg and effector T cell responses, we analyzed the expression of *TLR*s (2, 4, 7, 8, 9) and immune checkpoint molecules (*CTLA-4*, *IDO-1*, *LAG3*, *PD-1*) in THP-1-derived macrophages 3 h after exposure to gDNA from *C. albicans*, *S. cerevisiae*, or *C. neoformans*. RT-qPCR results were normalized to GAPDH expression in unstimulated macrophages (Figure 5).

At the transcriptional level, CalbG and ScerG induced broadly similar patterns, characterized by downregulation of *TLR*s, especially *TLR-4* and *TLR-9*. In contrast, CrypG strongly upregulated *TLR-4* and *TLR-9*, consistent with robust Teff activation. Checkpoint gene expression was species-specific: *CTLA-4* was notably increased by ScerG, *IDO-1* was upregulated by CalbG, and *LAG3* remained largely unchanged across all treatments. The elevated checkpoint gene expression in *C. albicans* and *S. cerevisiae* may explain their modest Teff responses and Breg response, which may contribute their more tolerogenic or commensal interactions with the host, contrasting with the proinflammatory TLR activation by *Cryptococcus* genomic DNA.

### 3.6. Species-Specific Engagement of Fungal DNA on cGAS–STING and TLR-9 Pathways

**Figure 6:**
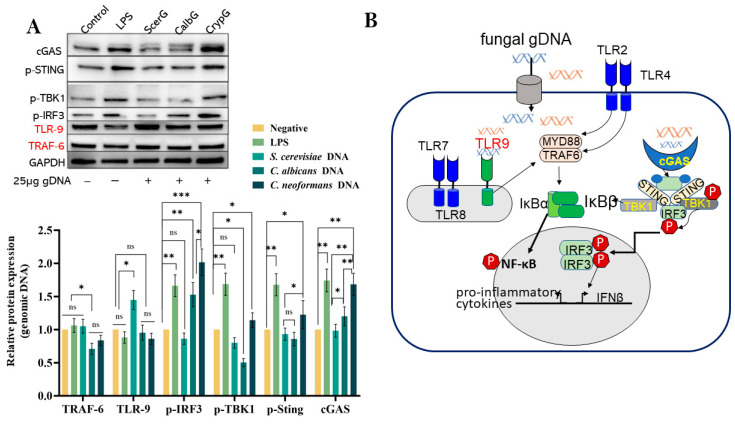
Differential activation of TLR-9–MyD88 and the cGAS—STING pathways by fungal DNAs in THP-1—derived macrophages. (**A**) Western blot analysis of protein expression in macrophages treated for 6 h with genomic DNA from *C. albicans* (CalbG), *S. cerevisiae* (ScerG), or *C. neoformans* (CrypG), compared with PBS and LPS controls. Blots were probed for TLR-9 and TRAF-6 (TLR-9–MyD88 axis) and for cGAS, p-STING, p-TBK1, and p-IRF3 (cGAS–STING axis). GAPDH served as a loading control. Each bar graph shows quantification from three independent blots. (**B**) Schematic illustration of the two DNA-sensing pathways converging at TBK1: the TLR-9–MyD88–TRAF6 axis driving NF-κB-dependent inflammatory responses, and the cGAS–STING cascade leading to p-IRF3-dependent transcriptional programs. Data are shown as mean ± SD from ImageJ analysis of three independent blots. (* *p* < 0.05, ** *p* < 0.01, *** *p* < 0.001, and ns denotes no significance).

At the protein level, we focused on two canonical DNA-sensing pathways in macrophages treated with fungal DNAs: the TLR-9–MyD88 axis and the cGAS–STING pathway. THP-1-derived macrophages were exposed to gDNA for 6 h, and protein expression was assessed by Western blot analysis normalized to GAPDH protein (Figure 6A). TLR-9 and TRAF-6 were used as activation markers of the TLR-9–MyD88 pathway, while cGAS, p-STING, p-TBK1, and p-IRF3 reflected activation of the cGAS–STING axis (Figure 6B).

As shown in Figure 6A, genomic DNA from all three yeasts induced comparable TLR-9 and TRAF-6 expression, with slightly higher TLR-9 in ScerG and slightly reduced TRAF-6 protein in CalbG compared to controls (*p* < 0.05). No other marked differences in TLR-9 signaling were observed between DNA samples and the LPS control. By contrast, the cGAS–STING pathway was strongly activated by *C. neoformans* DNA, as evidenced by robust cGAS expression and phosphorylation of STING, TBK1, and IRF3. In *C. albicans* DNA–treated cells, cGAS–STING activation was milder, characterized by modest p-IRF3 induction and a distinctive two-band cGAS pattern. This upper band, absent in unstimulated controls, may reflect a post-translationally modified or ligand-bound form of cGAS in CalbG condition, potentially linked to cGAMP binding [46]. ScerG produced the weakest cGAS–STING activation, consistent with its lower overall immunostimulatory activity.

Taken together with the mRNA data (Figure 5), these findings suggest that while Treg induction is a shared outcome across fungal DNA sources, the upstream APC activation pathways diverge. Pathogenic *C. neoformans* DNA strongly engages proinflammatory TLR-9 and cGAS–STING signaling, whereas *C. albicans* and *S. cerevisiae* DNA induce checkpoint gene expression and weak IRF3/TBK1 phosphorylation, promoting immune tolerance.

## 4. Discussion

Despite increasing recognition of fungi in the gut as potent modulators of host immunity, most studies have focused primarily on cell surface components—such as mannoproteins and glucans—that engage pattern recognition receptors like TLR-2, TLR-4, and Dectin-1, -2, and -3. In contrast, the immunological roles of fungal DNA remain largely underexplored, particularly regarding cytosolic sensing and regulatory immune responses. Our study seeks to examine how purified fungal genomic DNA from commensal *C. albicans*, *S. cerevisiae*, and pathogenic *C. neoformans* differentially shape adaptive T cell polarization, focusing on Treg versus effector T cell responses.

Fungal DNA is recognized and internalized by macrophages through various pattern recognition receptors. These effects could occur, at least in part, through uptake pathways such as scavenger receptors, including CD36 and macrophage scavenger receptor 1 (MSR1) [47], or through calcium channel-mediated endocytosis [48,49]. These receptors have been implicated in the recognition and uptake of fungal pathogens and may similarly contribute to the internalization of fungal DNA, although direct evidence remains scarce. Upon internalization, DNA is processed in endosomal compartments by TLR-9, which preferentially recognizes unmethylated CpG motifs commonly found in bacterial and mitochondrial DNA [50,51], thereby promoting immune activation. Three major signaling pathways govern DNA-induced immune activation: the cyclic GMP-AMP synthase (cGAS)–stimulator of interferon genes (STING) pathway, the TLR-9/MyD88 pathway, and the AIM2/inflammasome pathway [18,52]. Activation of the cGAS–STING or TLR-9 pathway leads to upregulation of type I interferons (IFNs), TNFα, IL-6, and other proinflammatory cytokines and chemokines, whereas stimulation of the inflammasome pathway results in elevated IL-1β and IL-18 levels. Dysregulation of these DNA-sensing pathways can result in persistent inflammatory signaling, contributing to the development of autoimmune diseases and cancer [5,53,54].

Using human CD4^+^ T cells and murine splenocytes, we show that fungal DNA can act as either a tolerogenic or inflammatory signal, depending on the species. *C. albicans* gDNA uniquely promotes Treg induction while preserving CD4^+^ T cell viability in an inflammatory milieu (IL-2 + TGF-β stimulation) or in splenocytes, suggesting that its DNA may help counterbalance T cell exhaustion and inflammation induced by fungal infection. DNA from *S. cerevisiae* induces similar Treg responses but generates fewer effector T cells, accompanied by higher checkpoint gene expression, likely reflecting its harmless commensal nature. In contrast, *C. neoformans* DNA elicits partial Treg responses and lacks checkpoint gene induction while strongly promoting Th1/Th17 and inflammatory cytokine responses, highlighting its capacity to drive effector programs. Together, these findings suggest that fungal genomic DNA conveys species-specific immune signatures that shape the balance between tolerance and inflammation.

To further understand the mechanistic basis for these divergent outcomes, we examine how fungal DNA influences key pathways involved in antifungal immunity in THP-1–derived macrophages. Our findings align with the well-documented interplay between Th17 and Treg cells, where both subsets are indispensable [55,56]: depletion of either IL-17 or Tregs increases susceptibility to oropharyngeal and systemic candidiasis [57,58]. Transcriptional analyses provide a framework for these divergent outcomes. At 3 h post-DNA treatment, *C. albicans* and *S. cerevisiae* gDNAs downregulated *TLR-4* and *TLR-9* while upregulating checkpoint molecules such as *IDO-1* and *CTLA-4*, consistent with an immune tolerance phenotype. In contrast, *C. neoformans* DNA strongly activates TLR-9 transcripts without appreciable checkpoint induction, aligning with a proinflammatory immune profile. These findings suggest that the species-specific regulation of TLRs and checkpoint genes by different fungal DNA may directly influence CD4^+^ T cell polarization toward different subsets and modulate inflammatory cytokine production, although the precise molecular mechanisms remain to be determined.

Notably, all three fungal gDNAs elicit measurable Th2 responses, regardless of their distinct effects on Treg and Th17 subsets. While Th2 immunity is not the primary focus of this study, this observation aligns with a previous report that both commensal and pathogenic fungi tend to bias host responses toward Th2-type programs [45], traditionally associated with defense against multicellular parasites. This baseline Th2 skewing may represent an evolutionary safeguard against fungal persistence at barrier sites, while also contributing to allergic sensitization under permissive conditions.

*TLR-8* upregulation by *S. cerevisiae* DNA (Figure 5) and the slightly increased TLR-9 protein level (Figure 6A)—not seen for *C. albicans* DNA in either case—are unusual and intriguing. We cannot exclude the possibility that this represents a species-specific effect, and whether TLR-8 contributes to immunotolerance requires further investigation. One recent study showed that fungal nucleic acids from *C. albicans* can stimulate neutrophil extracellular trap (NET) formation via TLR8- and TLR9-dependent pathways [59]. TLR-8 is classically considered an ssRNA sensor, primarily recognizing viral or bacterial single-stranded RNA in endosomes. Several factors may be associated with this upregulation, including minor contamination with GU-rich RNA in the gDNA preparation, the formation of RNA-DNA hybrids or G-quadruplexes during endosomal processing that mimic TLR-8 and TLR-9 ligands [60], and the naturally hypomethylated state of *S. cerevisiae* DNA [61]. Therefore, while the precise mechanism remains unclear, it is plausible that the persistent TLR-8 upregulation associated with *S. cerevisiae* reflects an evolved mechanism to fine-tune immune responses without triggering strong inflammation, consistent with its status as a relatively harmless commensal compared with *C. albicans*.

This study is limited by its reliance on in vitro and ex vivo systems, which cannot fully capture the complexity of host–fungus interactions in vivo, including the contributions of tissue-resident antigen-presenting cells, microbiome interactions, and systemic cytokine networks. Moreover, the concentrations of DNA used may not precisely reflect exposure levels during commensal colonization versus invasive infection.

Despite these limitations, our findings reveal a species-specific divergence in Treg versus effector T cell programming by fungal DNA: *C. albicans* promotes a checkpoint-driven tolerogenic axis that balances antifungal defense with immune tolerance, whereas *C. neoformans* bypasses this restraint, amplifying effector responses. These results underscore the potential of fungal DNA as a modulatory signal shaping the equilibrium between tolerance and immunopathology.

Future studies should validate these mechanisms in vivo, dissect the relative contributions of TLR-9 and the cGAS–STING pathways, and explore how fungal DNA sensing influences clinical outcomes in fungal diseases.

## 5. Conclusions

Our study suggests that fungal genomic DNA can act as an active immunomodula-tor, influencing the balance between tolerance and inflammation in a species-specific manner. While commensal *C. albicans* and *S. cerevisiae* DNA appear to favor checkpoint induction and regulatory responses, pathogenic *C. neoformans* DNA engages DNA-sensing pathways more aggressively, promoting inflammatory immunity. These findings provide preliminary insights into fungal–host interactions and indicate that fungal DNA may contribute to the immune equilibrium that distinguishes commensalism from pathogenicity. Future in vivo studies will be important to validate these observations and explore their physiological relevance.

## Data Availability

Data are contained within the article and Supplementary Materials.

## Supplementary Materials

The following supporting information can be downloaded at https://www.mdpi.com/article/10.3390/jof11110760/s1: Figure S1: Schematic gating strategy for Treg cells from human PBMCs; Figure S2: Schematic gating strategy for Treg cells from murine splenocytes; Table S1: Primer sets for real-time qPCR of THP-1-derived macrophages.

## Abbreviations

*CalbG*	*Candida albicans* nuclear genomic DNA
*cGAS-STING*	cyclic GMP–AMP synthase–stimulator of interferon genes
*CrypG*	*Cryptococcus neoformans* nuclear genomic DNA
*DAMPs*	damage-associated molecular patterns
*gDNA*	genomic DNA
*IkBa*	inhibitor of nuclear factor kappa B alpha
*IkBb*	inhibitor of nuclear factor kappa B beta
*IRF-3*	interferon regulatory factor 3
*NF-κB*	nuclear factor kappa-light-chain-enhancer of activated B cells
*NET*	neutrophil extracellular trap
*PAMPs*	pathogen-associated molecular patterns
*PBMC*	peripheral blood mononuclear cell
*PRRs*	pattern recognition receptors
*ScerG*	*Saccharomyces cerevisiae* nuclear genomic DNA
*TLRs*	toll-like receptors
*TRAF-6*	TNF receptor-associated factor 6

## References

[1] Urban C.F., Reichard U., Brinkmann V., Zychlinsky A. (2006). Neutrophil extracellular traps capture and kill *Candida albicans* yeast and hyphal forms. Cell Microbiol..

[2] Huang H., Li M., Luo M., Zheng J., Li Q., Wang X., Liu Y., Li D., Xi L., Liu H. (2024). Neutrophil extracellular traps (NETs) and Th-2 dominant immune responses in chronic granulomatous chromoblastomycosis. Med. Mycol..

[3] Hasim S., Coleman J.J. (2019). Targeting the fungal cell wall: Current therapies and implications for development of alternative antifungal agents. Future Med. Chem..

[4] Rizzo J., Wong S.S.W., Gazi A.D., Moyrand F., Chaze T., Commere P.H., Novault S., Matondo M., Péhau-Arnaudet G., Reis F.C.G. (2021). *Cryptococcus* extracellular vesicles properties and their use as vaccine platforms. J. Extracell Vesicles..

[5] Zhang R., Wiederhold N., Calderone R., Li D. (2024). Biofilm formation in clinical isolates of *Fusarium*. J. Fungi.

[6] Kim J., Pena J.V., McQueen H.P., Kong L., Michael D., Lomashvili E.M., Cook P.R. (2024). Downstream STING pathways IRF3 and NF-κB differentially regulate CCL22 in response to cytosolic dsDNA. Cancer Gene Ther..

[7] Häcker G., Redecke V., Häcker H. (2002). Activation of the immune system by bacterial CpG-DNA. Immunology.

[8] Pisetsky D.S. (1996). Immune activation by bacterial DNA: A new genetic code. Immunity.

[9] Briard B., Fontaine T., Kanneganti T.D., Gow N.A.R., Papon N. (2021). Fungal cell wall components modulate our immune system. Cell Surf..

[10] de Assis L.J., Bain J.M., Liddle C., Leaves I., Hacker C., Peres da Silva R., Yuecel R., Bebes A., Stead D., Childers D.S. (2022). Nature of β-1,3-glucan-exposing features on *Candida albicans* cell wall and their modulation. mBio.

[11] Lionakis M.S., Drummond R.A., Hohl T.M. (2023). Immune responses to human fungal pathogens and therapeutic prospects. Nat. Rev. Immunol..

[12] Loh J.T., Lam K.P. (2023). Fungal infections: Immune defense, immunotherapies and vaccines. Adv. Drug Deliv. Rev..

[13] Elsegeiny W., Marr K.A., Williamson P.R. (2018). Immunology of cryptococcal infections: Developing a rational approach to patient therapy. Front. Immunol..

[14] Chadeganipour M., Shadzi S., Mohammadi R. (2021). Fungal infections among psoriatic patients: Etiologic agents, comorbidities, and vulnerable population. Autoimmune Dis..

[15] Mazziotta C., Tognon M., Martini F., Torreggiani E., Rotondo J.C. (2023). Probiotics mechanism of action on immune cells and beneficial effects on human health. Cells.

[16] Li D., Cruz I., Peltak S.N., Foley P.L., Bellanti J.A. (2025). Methylated CpG ODNs from *Bifidobacterium longum* subsp. *infantis* modulate treg induction and suppress allergic response in a murine model. Int. J. Mol. Sci..

[17] Prescott S., Saffery R. (2011). The role of epigenetic dysregulation in the epidemic of allergic disease. Clin. Epigenetics..

[18] Briard B., Place D.E., Kanneganti T.D. (2020). DNA sensing in the innate immune response. Physiology.

[19] Liao W., Du C., Wang J. (2020). The cGAS-STING pathway in hematopoiesis and its physiopathological significance. Front. Immunol..

[20] Li D., Cheng J., Zhu Z., Catalfamo M., Goerlitz D., Lawless O.J., Tallon L., Sadzewicz L., Calderone R., Bellanti J.A. (2020). Treg-inducing capacity of genomic DNA of *Bifidobacterium longum* subsp. *infantis*. Allergy Asthma Proc..

[21] Li D., Sorkhabi S., Cruz I., Foley P.L., Bellanti J.A. (2025). Studies of methylated CpG ODN from *Bifidobacterium longum* subsp. *infantis* in a murine model: Implications for treatment of human allergic disease. Allergy Asthma Proc..

[22] Gu J., Lu L., Chen M., Xu L., Lan Q., Li Q., Liu Z., Chen G., Wang P., Wang X. (2014). TGF-β-induced CD4+Foxp3+ T cells attenuate acute graft-versus-host disease by suppressing expansion and killing of effector CD8+ cells. J. Immunol..

[23] Wahl S.M., Chen W. (2005). Transforming growth factor-beta-induced regulatory T cells referee inflammatory and autoimmune diseases. Arthritis Res. Ther..

[24] Hoffmann P., Eder R., Boeld T.J., Doser K., Piseshka B., Andreesen R., Edinger M. (2006). Only the CD45RA+ subpopulation of CD4+CD25high T cells gives rise to homogeneous regulatory T-cell lines upon in vitro expansion. Blood.

[25] Bellanti J.A., Li D. (2021). Treg cells and epigenetic regulation. Adv. Exp. Med. Biol..

[26] Sekiya T., Nakatsukasa H., Lu Q., Yoshimura A. (2016). Roles of transcription factors and epigenetic modifications in differentiation and maintenance of regulatory T cells. Microbes Infect..

[27] Polansky J.K., Schreiber L., Thelemann C., Ludwig L., Krüger M., Baumgrass R., Cording S., Floess S., Hamann A., Huehn J. (2010). Methylation matters: Binding of Ets-1 to the demethylated Foxp3 gene contributes to the stabilization of Foxp3 expression in regulatory T cells. J. Mol. Med..

[28] Tsuji-Takayama K., Suzuki M., Yamamoto M., Harashima A., Okochi A., Otani T., Inoue T., Sugimoto A., Toraya T., Takeuchi M. (2008). The production of IL-10 by human regulatory T cells is enhanced by IL-2 through a STAT5-responsive intronic enhancer in the IL-10 locus. J. Immunol..

[29] Gunsalus K.T., Tornberg-Belanger S.N., Matthan N.R., Lichtenstein A.H., Kumamoto C.A. (2015). Manipulation of host diet to reduce gastrointestinal colonization by the opportunistic pathogen *Candida albicans*. mSphere.

[30] Neville B.A., d’Enfert C., Bougnoux M.E. (2015). *Candida albicans* commensalism in the gastrointestinal tract. FEMS Yeast Res..

[31] Kumamoto C.A., Gresnigt M.S., Hube B. (2020). The gut, the bad and the harmless: *Candida albicans* as a commensal and opportunistic pathogen in the intestine. CurrOpin Microbiol..

[32] d’Enfert C., Kaune A.K., Alaban L.R., Chakraborty S., Cole N., Delavy M., Kosmala D., Marsaux B., Fróis-Martins R., Morelli M. (2021). The impact of the Fungus-Host-Microbiota interplay upon *Candida albicans* infections: Current knowledge and new perspectives. FEMS Microbiol. Rev..

[33] Bacher P., Hohnstein T., Beerbaum E., Röcker M., Blango M.G., Kaufmann S., Röhmel J., Eschenhagen P., Grehn C., Seidel K. (2019). Human anti-fungal th17 immunity and pathology rely on cross-reactivity against *Candida albicans*. Cell.

[34] Pérez J.C. (2021). Fungi of the human gut microbiota: Roles and significance. Int. J. Med. Microbiol..

[35] Scheffold A., Bacher P., LeibundGut-Landmann S. (2020). T cell immunity to commensal fungi. CurrOpin Microbiol..

[36] Kong H.H., Segre J.A. (2020). Cultivating fungal research. Science.

[37] Zhang J., Feng Y., Li D., Shi D. (2024). Fungal influence on immune cells and inflammatory responses in the tumor microenvironment (Review). Oncol. Lett..

[38] Lim C.S., Tung C.H., Rosli R., Chong P.P. (2008). An alternative *Candida* spp. cell wall disruption method using a basic sorbitol lysis buffer and glass beads. J. Microbiol. Methods..

[39] Li D., Cruz I., Sorkhabi S., Foley P.L., Wagner J., Bellanti J.A. (2025). Dose-response studies of methylated and nonmethylated CpG ODNs from *Bifidobacterium longum* subsp. *infantis* for optimizing Treg cell stimulation. Allergy Asthma Proc..

[40] Li D., Cheng J., Calderone R., Bellanti J.A. (2022). Measurements of Treg cell induction by *Candida albicans* DNA using flow cytometry. Methods Mol. Biol..

[41] Janes K.A. (2015). An analysis of critical factors for quantitative immunoblotting. Sci. Signal..

[42] McAleer J.P., Vella A.T. (2008). Understanding how lipopolysaccharide impacts CD4 T-cell immunity. Crit. Rev. Immunol..

[43] Bao M., Ehexige E., Xu J., Ganbold T., Han S., Baigude H. (2021). Oxidized curdlan activates dendritic cells and enhances antitumor immunity. Carbohydr. Polym..

[44] Yi E.J., Kim Y.I., Song J.H., Ko H.J., Chang S.Y. (2023). Intranasal immunization with curdlan induce Th17 responses and enhance protection against enterovirus 71. Vaccine.

[45] Zheng Y., Dang E.V. (2023). Novel mechanistic insights underlying fungal allergic inflammation. PLoS Pathog..

[46] Zierhut C., Funabiki H. (2020). Regulation and consequences of cGAS Activation by self-DNA. Trends Cell Biol..

[47] Means T.K. (2010). Fungal pathogen recognition by scavenger receptors in nematodes and mammals. Virulence.

[48] Husna N., Aiba T., Fujita S.I., Saito Y., Shiba D., Kudo T., Takahashi S., Furukawa S., Muratani M. (2024). Release of CD36-associated cell-free mitochondrial DNA and RNA as a hallmark of space environment response. Nat. Commun..

[49] Asif A., Mohsin H., Tanvir R., Rehman Y. (2017). Revisiting the mechanisms involved in calcium chloride induced bacterial transformation. Front. Microbiol..

[50] Elias F., Flo J., Lopez R.A., Zorzopulos J., Montaner A., Rodriguez J.M. (2003). Strong cytosine-guanosine-independent immunostimulation in humans and other primates by synthetic oligodeoxynucleotides with PyNTTTTGT motifs. J. Immunol..

[51] Ingelsson B., Söderberg D., Strid T., Söderberg A., Bergh A.C., Loitto V., Lotfi K., Segelmark M., Spyrou G., Rosén A. (2018). Lymphocytes eject interferogenic mitochondrial DNA webs in response to CpG and non-CpG oligodeoxynucleotides of class C. Proc. Natl. Acad. Sci. USA.

[52] Chen Q., Sun L., Chen Z. (2016). Regulation and function of the cGAS–STING pathway of cytosolic DNA sensing. Nat. Immunol..

[53] Sekigawa I., Okada M., Ogasawara H., Kaneko H., Hishikawa T., Hashimoto H. (2003). DNA methylation in systemic lupus erythematosus. Lupus.

[54] Chen B., Sun L., Zhang X. (2017). Integration of microbiome and epigenome to decipher the pathogenesis of autoimmune diseases. J. Autoimmun..

[55] Whibley N., Gaffen S.L. (2014). Brothers in arms: Th17 and Treg responses in *Candida albicans* immunity. PLoS Pathog..

[56] Hernández-Santos N., Gaffen S.L. (2012). Th17 cells in immunity to *Candida albicans*. Cell Host Microbe..

[57] Pandiyan P., Conti H.R., Zheng L., Peterson A.C., Mathern D.R., Hernandez-Santos N., Edgerton M., Gaffen S.L., Lenardo M.J. (2011). CD4(+)CD25(+)Foxp3(+) regulatory T cells promote Th17 cells in vitro and enhance host resistance in mouse *Candida albicans* Th17 cell infection model. Immunity.

[58] Huang W., Na L., Fidel P.L., Schwarzenberger P. (2004). Requirement of interleukin-17A for systemic anti-*Candida albicans* host defense in mice. J. Infect. Dis..

[59] Smolarz M., Zawrotniak M., Satala D., Rapala-Kozik M. (2021). Extracellular nucleic acids present in the *Candida albicans* biofilm trigger the release of neutrophil extracellulart. Front. Cell Infect. Microbiol..

[60] Forsbach A., Samulowitz U., Völp K., Hofmann H.P., Noll B., Tluk S., Schmitz C., Wader T., Müller C., Podszuweit A. (2011). Dual or triple activation of TLR7, TLR8, and/or TLR9 by single-stranded oligoribonucleotides. Nucleic Acid. Ther..

[61] Nai Y.S., Huang Y.C., Yen M.R., Chen P.Y. (2021). Diversity of Fungal DNA Methyltransferases and their association with DNA methylation patterns. Front. Microbiol..

