# Supplementary material for: Divergent Immunomodulatory Roles of Fungal DNA in Shaping Treg and Inflammatory Responses
Source: J Fungi (Basel). 2025 Oct 22;11(11):760. doi: 10.3390/jof11110760 (PMC12653120; doi:10.3390/jof11110760)
Supplement: Supplementary file 1 [file jof-11-00760-s001.zip › jof-3917645-supplementary.pdf]

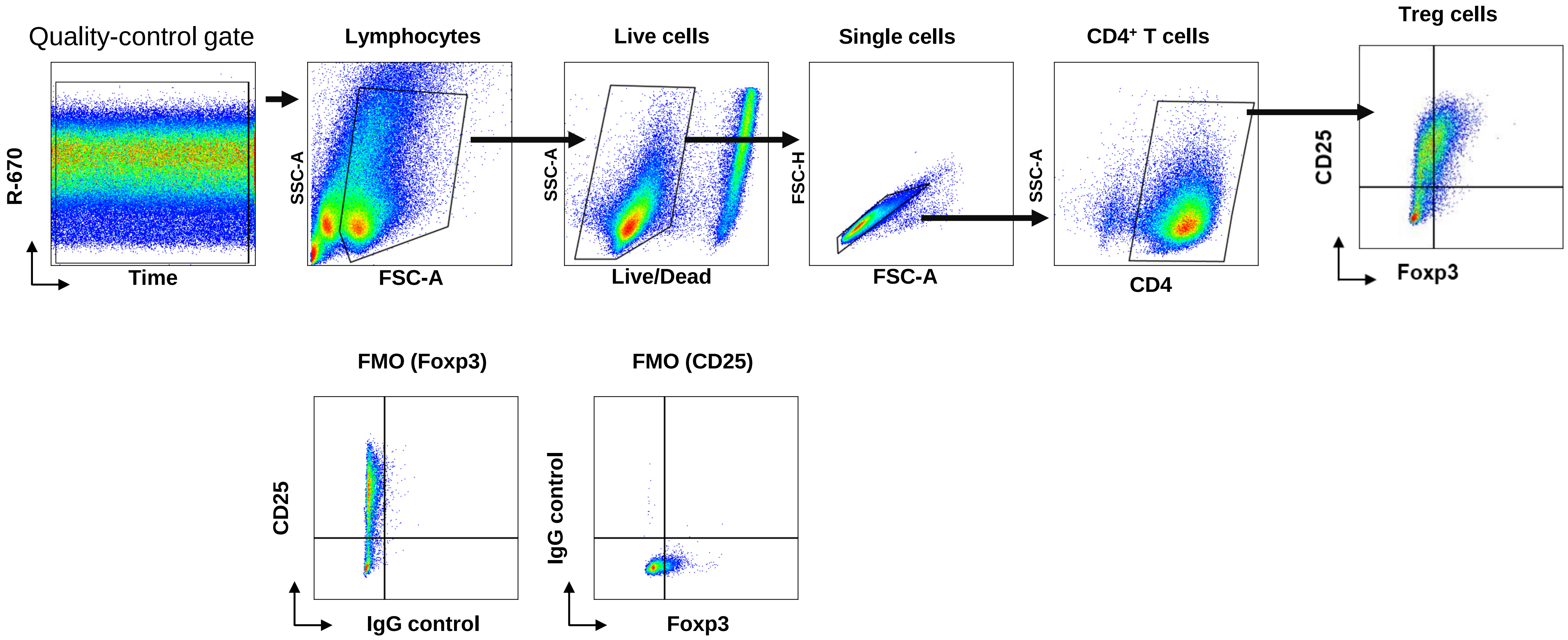

Figure S1: Gating strategy for identifying human $CD4^+$ Treg cells. Human $CD4^+$ T cell isolation is described in the Materials and Methods section. Sequential gates include R670/Time (viability dye vs. acquisition time; quality-control gate), SSC-A/FSC-A (lymphocyte gate), Live/Dead vs. SSC-A (live cell selection), FSC-H/FSC-A (single-cell discrimination), SSC-A/CD4 ($CD4^+$ cell identification), and $Foxp3^+/CD25^+$ (Treg gating). Corresponding FMOs (CD25/IgG control and IgG control/Foxp3) were used, with isotype IgG controls included to account for nonspecific antibody binding and to define accurate gating thresholds for $Foxp3^+$ and $CD25^+$ cells.

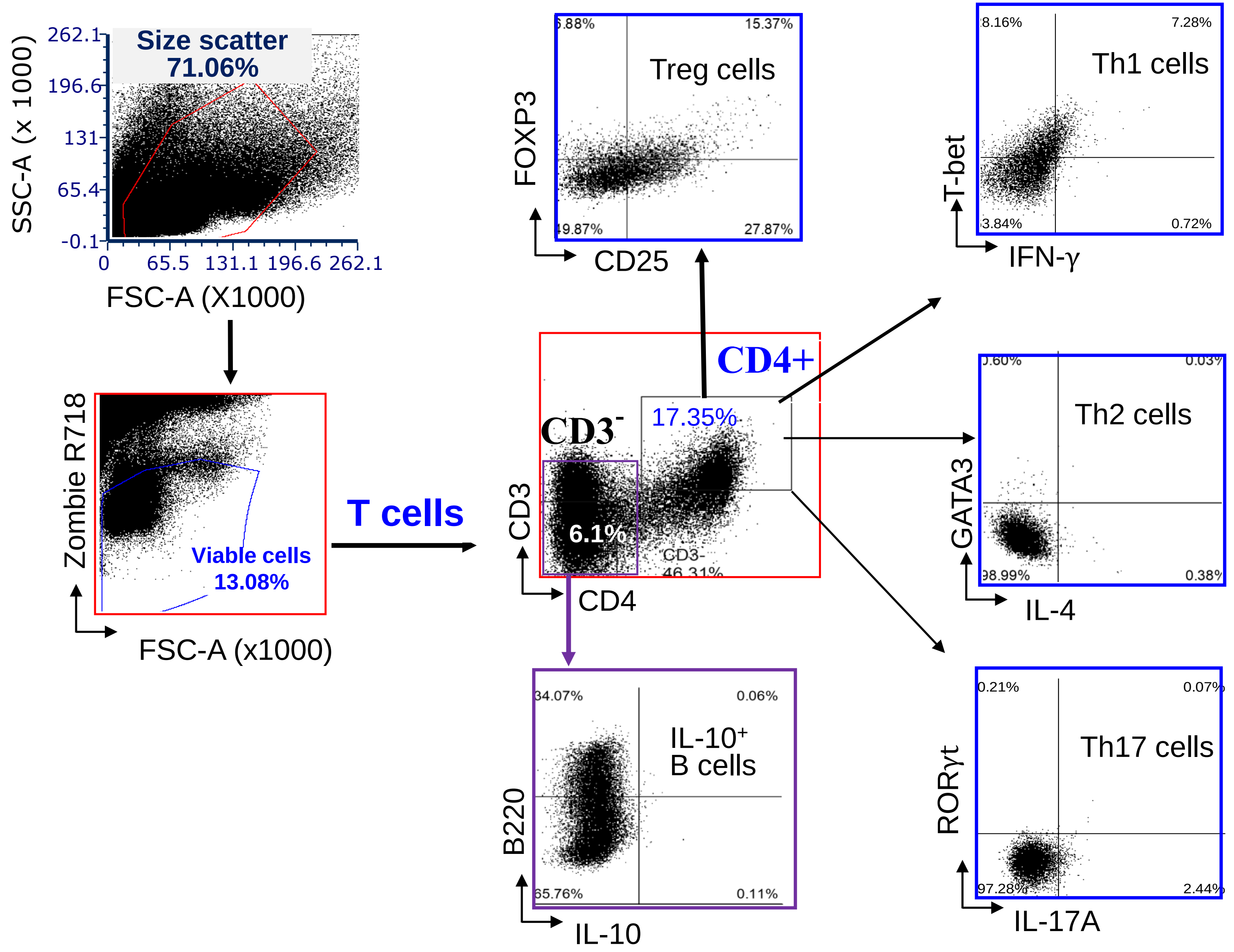

Figure S2: Schematic gating strategy for identifying Treg and effector T cell subsets from murine splenocytes. Sequential gates include SSC-A/FSC-A, Zombie R718 (viability dye; used to exclude dead cells), and CD3^{+}CD4^{+} (selection of CD4^{+} T cells). CD4^{+} cells were subsequently gated into functional subsets, including Th1 (IFN-γ^{+}T-bet^{+}), Th2 (IL-4^{+}GATA3^{+}), and Th17 (IL-17A^{+}RORγt^{+}) populations. IL-10^{+} B cells (IL-10^{+}, B220^{+}) gated from CD3^{-} cells. Gates were defined using appropriate fluorescence minus one (FMO) and isotype controls.

Table S1: Primer sets for real-time qPCR of THP-1–derived macrophages.

Gene name	Sequence 5’-3’
GAPDH-F	GGTGGTCTCCTCTGACTTCAACA
GAPDH-R	GTTGCTGTAGCCAAATTCGTTGT
TLR9-F	GCATCTCGCAGGCAGTCAATGG
TLR9-R	CCGTGAATGAGTGCTCGTGGTAG
TLR8-F	GGAGCCAGTGTTACAGCATTCTCAG
TLR8-R	GCCTTCTGCCTTCGGGTTGTC
TLR7-F	ACCAACTGACCACTGTCCCTGAG
TLR7-R	ATCGCAACTGGAAGGCATCTTGTAG
TLR2-F	CTACCAGATGCCTCCCTCTTACCC
TLR2-R	ACCAGCTTCCAAAGTCTTCAGTGTG
TLR4-F	GCTCTTGGTGGAAGTTGAACGAATG
TLR4-R	CAAGCACACTGAGGACCGACAC
IDO1-F	GCCCTTCAAGTGTTTCACCAAATCC
IDO1-R	GGGTTGCCTTTCCAGCCAGAC
CTLA4-F	TGGACACGGGACTCTACATCTGC
CTLA4-R	TAAATCTGGGTTCCGTTGCCTATGC
LAG3-F	GCCACCTCCTGCTGTTTCTCATC
LAG3-R	GGTCGCCACTGTCTTCTCCAAAG
PDCD1-F	GTGCCTGTGTTCTCTGTGGACTATG
PDCD1-R	TGAGGTGCCCATTCCGCTAGG
